# Cardiovascular implantable electronic devices: a review of the dangers and difficulties in MR scanning and attempts to improve safety

**DOI:** 10.1007/s13244-017-0556-3

**Published:** 2017-06-17

**Authors:** Pei Ghim Poh, Charlene Liew, Colin Yeo, Le Roy Chong, Andrew Tan, Angeline Poh

**Affiliations:** 1 Department of Radiology, Changi General Hospital, 2 Simei Street 3, Singapore, 529889 Singapore; 2 Singhealth Radiology Residency, 167 Jalan Bukit Merah #17-10 Tower 5, Singapore, 150167 Singapore; 3 Department of Cardiology, Changi General Hospital, 2 Simei Street 3, Singapore, 529889 Singapore

**Keywords:** Equipment safety, Equipment design, Magnetic resonance imaging, Pacemaker, Artificial, Physics

## Abstract

**Abstract:**

An increasing number of patients are being treated with cardiovascular implantable electronic devices (CIEDs), many of which are MR conditional. There is a lack of literature on the safe scanning of MR conditional CIEDs. This review article discusses MR imaging safety in patients with implanted CIEDs. Guidelines on safe use and indications of imaging patients with MR conditional CIEDs are described, followed by a pictorial essay of the radiographic features of these devices. We also discuss the challenges of monitoring the patient in the MR environment, advances in MRI conditional imaging of devices, availability, limitations and workflow including vendor-specific and other collaborative efforts to simplify the scanning process. Radiologists must be able to facilitate the safe utilization of MR imaging in patients who have CIEDs. A thorough knowledge of the hazards of imaging non-MR compatible devices is required as well as knowing how to correctly identify and manage the imaging of patients with MR conditional CIEDs. Finally, we propose steps required to facilitate the safe scanning of patients with MR conditional CIEDs adopted in our institution and a contingency plan in the event that an inadvertent MR scan of a patient with a MRI unsafe CIED should occur.

***Main Messages*:**

• *Risks of MR imaging in patients who have CIEDs have been worked around.*

• *There are many technical limitations in enabling safe MR scanning of CIEDs.*

• *Radiological identification of MRI-conditional status of CIEDs is useful.*

• *Standardizing conditions for safe MRI scanning is important.*

• *We offer example algorithms for facilitating safe MRI scanning of CIEDs.*

## Introduction

Magnetic resonance imaging (MRI) has steadily increased in use worldwide [1]. We believe that there is a foreseeable increase in the number of patients with cardiovascular implantable electronic devices (CIEDs) who will require a MRI. An estimated 75% of patients with CIEDs will have an indication requiring MRI during their lifetime [ 2].

This review article discusses the safe use of MRI in patients who have implanted CIEDs. Radiologists should have a basic grasp of the principles behind the re-design and engineering of these devices, once considered to be an absolute contraindication to scanning.

The known hazards of MRI unsafe CIEDs are described, followed by guidelines on safe use, indications and limitations of imaging patients with MR conditional CIEDs, including a brief pictorial essay of the radiographic features of these devices.

Finally, we also offer steps to facilitate the safe scanning of patients with MR conditional CIEDs in the form of a proposed guideline, which was adopted in our institution, and a contingency plan if an inadvertent MR scan of a patient with a MRI unsafe CIED should occur. These have been ratified by the authors’ institutional medical board review process and have been implemented successfully at the time of writing.

### What are MRI safe devices?

Implants, devices or materials should routinely undergo evaluation prior to a MRI procedure if known, and are categorized as safe, conditional or unsafe [3].

Safe devices are safe within the MR environment. The object is usually made from non-ferromagnetic components. There are no MR safe cardiovascular implantable devices at the time of writing.

Conditional devices are safe for the patient undergoing MRI only if specific conditions are met. This is usually often due to the presence of a weakly ferromagnetic component in the implantable device. Current recommendations support safe scanning at 1.5 T although several studies with scanning at 3 T exists.

Unsafe devices pose potential risk(s) to an individual in the MR environment, for which the physical concepts are discussed below.

## Hazards of imaging MRI unsafe cardiovascular devices

A cardiac pacemaker system is composed of leads and a pulse generator. Within the pulse generator are connectors, circuitry and a battery which may be ferromagnetic. This results in interactions with the magnetic field or RF pulse in MR environments [1, 2].

**Fig. 1:**
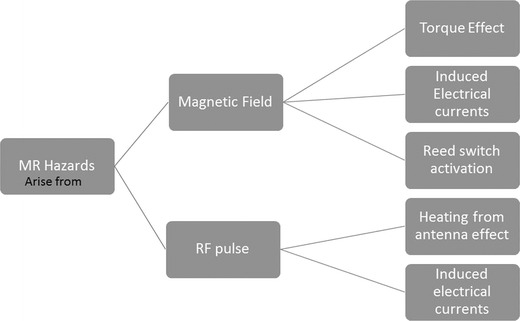
MR hazards which pose a risk to patients with MRI unsafe compatible CIEDs

Magnetic field interactions include torque effect, induced electrical currents and reed/Hall sensor switch activation. RF pulse effects may also cause induced electrical currents and the antenna effect. All reported MRI-induced burns originate from electromagnetic induction or the antenna effect. Previous studies [4, 5] identifying issues regarding such interactions have formed the basis for the engineering of MR conditional devices (See Fig. 1).

### Torque effect

The presence of ferromagnetic materials in the device may lead to movement or vibration in a MR environment. The forces are directly related to the shape and amount of the ferromagnetic material as well as the field strength and its location within the magnetic field [6]. The torque induced by the magnetic field may result in movement of internal components or dislodgement of its components. Prospective studies on conventional and MRI-conditional pacemakers have not demonstrated significant adverse effects [7, 8].

### Induced electrical currents

Electromagnetic energy may conduct through the pacing system due to a change in magnetic flux, sources of which include the pulsed radiofrequency energy from a surface coil [9] and the time-varying magnetic fields used for the spatial localization of signals [10].

The transfer of magnetic radiofrequency energy to heat and electrical energy depends on the pulse sequence parameters, the whole-body averaged and specific absorption rates (SAR), spatial relation and orientation with regards to the coil and configuration of the lead (composition, length and orientation) [6].

Induced electrical currents may mimic intrinsic cardiac activity. This may result in oversensing (where activity is interpreted as ventricular tachycardia) or undersensing (failure to sense native cardiac activity) resulting in an inappropriate high rate or an inhibition of pacing in CIEDs. In implantable cardioverter defibrillators (ICDs), this may result in inappropriate shock therapy [3]. Heating may also cause direct tissue damage such as oedema and formation of scar tissue at the lead-tissue interface [1, 11].

### Antenna effect

Another mechanism, which can result in heating, occurs when a wire of appropriate length is exposed to RF frequency and acts as an antenna. The amplitude of this effect is maximized at the resonant length of the antenna, generating electromagnetic oscillation, mainly at the antinodes or lead tips [12, 13]. This results in heat generation at the ends of the wire.

Subsequently, resultant sensing or capture threshold changes occur and may result in rapid pacing or loss of signal capture [10, 14], potentially causing inappropriate inhibition of demand pacing or tachycardia therapies.

### Reed switch activation

**Fig. 2:**
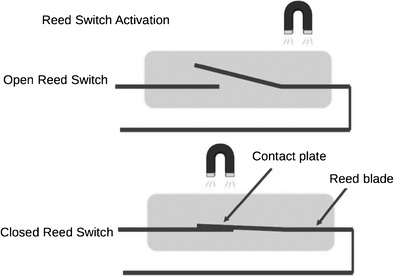
Diagram shows reed switch activation by a magnetic field

Older devices have a magnetically-activated reed switch, consisting of two metal strips in a glass capsule and can be activated or inactivated by an external magnetic field. Activation or deactivation depends on the orientation of the switch in relation to the static magnetic field (See Fig. 2).

When kept in open/closed positions, the CIED may also permanently disable tachycardia therapies or result in asynchronous pacing and this may be life-threatening in patients with recent myocardial infarction, hypoxemia or major electrolyte imbalance. Activation of the reed switch results in a preset pacing rate and may theoretically induce VF or aggravate myocardial ischemia. There may also be accelerated battery depletion [3, 15].

## Engineering CIEDs to make them MR conditional

**Fig. 3:**
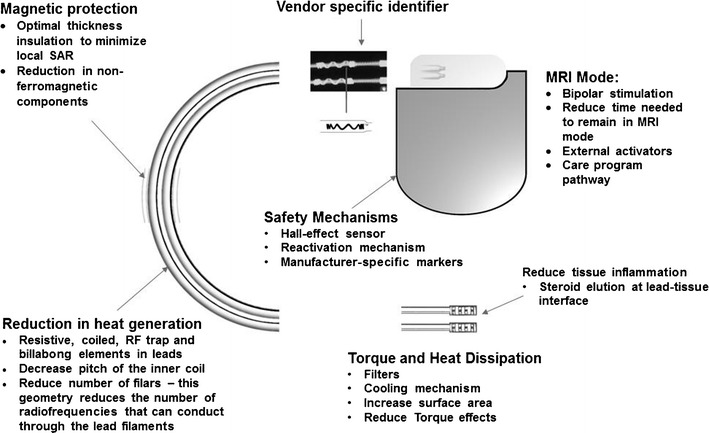
Schematic diagram details the various components of CIEDs, which have been re-engineered to make these MR-conditional

Almost all hazards related to CIEDs in MR environments are due to the presence of ferromagnetic content. There are two main ways to reduce magnetic field interaction. Firstly, through the minimization of ferromagnetic content of the generator and leads. This limits the choice of material which should be conductive, durable and biocompatible. The second method is to prevent the ferromagnetic content from interacting with the magnetic field, which can be achieved by using a lower magnetic field strength or by modification of the lead design. MRI-compatible leads can be identified by their vendor-specific identifier which will be covered in a separate section (See Fig. 3).

### Lead modification

Any wire in contact with soft tissue can result in tissue damage from heating and thus, insulation is required. However, as insulation thickness increases, local SAR also increases, resulting in increased heating at the tip. However, with specially designed leads, heating can be limited to below 1–2 degrees Celsius. An example of lead design changes in a MR conditional lead is a change in the pitch of the inner coil. Other methods include having fewer coiled filars, increasing the number of winding turns and, therefore, increasing lead inductance. This limits the radiofrequencies that can resonate through the lead filaments [16].

The use of bipolar sensing and low-pass filters reduces conducted and radiated interference. Additionally, feed-through capacitor filters are utilized to prevent electromagnetic induction from a wide range of frequencies [10].

### Lead-tissue interface

Whilst most of the lead is insulated, a lead-tissue interface allows bare wire to be in contact with soft tissue. In some animal models, noise or loss of capture in tips was noted after being exposed to MRI [17]. It was found that there was an increase in the lead tip temperature as well as oedema at the lead-tissue interface. Steroid-elution technology is one of the methods used to try to reduce inflammation at site of contact and to prevent threshold changes to produce consistent optimal threshold behaviour [18].

The use of titanium nitride, a biocompatible alloy, to coat electrodes has also been shown to avoid interference caused by noise by shifting the frequency of the band pass towards the lower frequency spectrum, thereby improving sensing performance [19]. Increased surface area of the lead tip in contact with soft tissue can result in reduced torque effects and a better cooling mechanism. For example, St Jude Medical employs the use of soft silicone pads at the tips, allowing a larger tip to be introduced through a smaller introducer due to the soft nature of the material, effectively reducing the tip pressure by approximately 50% in their test reports [20]. Allowing 6 weeks to elapse post-implantation allows better wound healing and fixation, thus reducing the effects of torque [9].

### Pulse generator shielding

Hermetically-sealed titanium or stainless steel cases are typically used to shield CIED generators and recently, nanomagnetic insulation has been used in leads to improve shielding from radiofrequency and time-varying gradient magnetic fields [10].

### Safety mechanisms

**Fig. 4:**
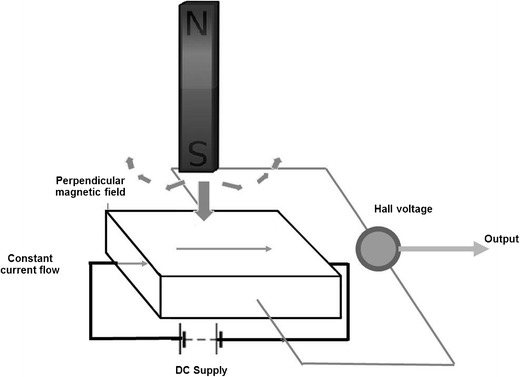
A Hall sensor varies its output voltage in response to a magnetic field

A magnet-activated switch such as a reed switch was designed to prevent adverse effects due to magnetic field interactions. This was considered unreliable and has now been replaced with Hall sensors. Hall sensors are based on the generation of voltage across an electrical conductor (See Fig. 4).

When the magnetic field is perpendicular to the direction of the conductor current flow, varied output voltage is generated in response to a magnetic field. Thus, this has a more predictable behaviour compared to a reed switch and can be ‘locked out’ when undergoing MRI. These switches function as transducers to trigger an electronic switch to ‘ON’ or ‘OFF’ when activated by a magnetic field. This halts inappropriate treatment during scanning [4, 5].

## Challenges of monitoring patients in the MR environment

A cardiologist should clear the patient for scanning and patients are supervised by a healthcare professional with appropriate training throughout the procedure, usually a cardiologist or pacemaker nurse. During scanning, sensing is stopped and diagnostics testing will be unavailable.

Pulse oximetry and ECG are standard devices that are integrated into the MRI room. These contain circuitry with specially designed ECG electrodes containing a minimal amount of metal. Standard ECG electrodes and leads may potentially burn the patient due to excessive heating. Artefacts may also affect ECG readings which are crucial for gated MRI procedures. Several techniques have been shown to reduce ECG artefacts, for example: positioning the line parallel to the magnetic field flux lines and placing limb electrodes close to each other [21, 22].

Back-up therapy should be available, some of which may have to be placed outside the MRI room. Other devices that can be modified include gurneys, oxygen tanks, stethoscopes, suction devices, infusion pumps and power injectors. Ventilators must also be modified due to the presence of mechanical switches or ferromagnetic components. Some ventilators only operate at a ‘safe’ distance from the MRI machine due to their ferromagnetic components [23]. These devices are MRI-conditional. Where temperature must be monitored, fluoroptic thermometry can be used [24].

Although no strict monitoring standard exists, a set of recommendations has been published in a joint statement by the Canadian Heart Rhythm Society and Canadian Association of Radiologists [25].

## MRI modes

MRI mode refers to changing CIED settings to accommodate an MRI environment such that oversensing and inappropriate therapy can be minimized and restoration of prescan program states and values are simplified [26]. MRI modes for MRI-conditional devices vary between different devices and manufacturer instructions should be followed. No standard approach exists between the different devices, but the concepts are akin to those in non-MRI-conditional devices.

It remains contradictory that in day-to-day radiology practice there have been clinical studies which show relative safety of scanning patients with CIEDs that are not MRI-conditional whilst most regulatory bodies continue to advocate scanning only MRI-conditional devices. This is may be revised in the future with an updated regulatory framework. In non-conditional devices, several parameters can be changed to make scanning safer. Patients with pacemakers are generally split between pacemaker-dependent patients and non-pacemaker dependent patients. ICD devices also require deactivation. These are clearly documented in the ESC guidelines and discussed below [27].

### Sensing only mode

This is often used in non-pacemaker-dependent patients. The pacemaker is programmed to off/sub-threshold outputs and the lead polarity is changed to bipolar [28].

### Asynchronous mode (DOO/VOO/AOO)

This is often used in pacemaker-dependent patients who are in a higher-risk group. In asynchronous mode, pacing occurs at a fixed rate which is well tolerated for short periods of time. There is an extremely low risk of developing VF during asynchronous pacing, and; hence, prolonged asynchronous pacing should be avoided [28].

### ICD temporary deactivation

ICD devices may falsely detect VT and subsequently deliver pacing, cardioversion or defibrillation therapies which may lead to actual arrhythmias. Although an in-built safety mechanism exists within the device, such as reed and Hall-sensor switches, deactivation remains a safer and predictable option.

Although scanning patients with non-conditional MRI devices carry additional risks, the actual rate of adverse events remains very low. A multicentre study (MagnaSafe Registry) has shown that in 1000 cases in which patients had a non-conditional MRI pacemaker and 500 cases in which patients had a non-conditional MRI ICD, only one ICD required immediate replacement, and the said device was not programmed appropriately before the MRI. Device parameter changes were common but only exceeded pre-specified thresholds in a small number of cases. Six cases of self-terminating atrial fibrillation/flutter and six cases of partial electrical reset were observed. There were no cases in which full electrical reset of the device

occurred [29].

At the time of this writing, the ESC strongly recommends against scanning patients with non-MRI-conditional pacemakers, especially those who are pacemaker-dependent. It is prudent to consider MRI as a last resort although perspectives may change with the release of additional data [30].

It is also important to note that pacemaker dependent patients who have an ICD are excluded from the Magnasafe Registry. No validated guideline exists for this subset of patients but asynchronous VOO mode with deactivation of ICD parameters appears to be a reasonable approach [31].

MRI of non-conditional CIEDs should not be considered as routine. Special precautions and patient selection is needed including requiring a cardiologist who has working knowledge to interrogate pacemakers and ICDs and where available, assistance of an industry device representative. The risk of lead removal for scanning is still probably higher than scanning non-MRI-conditional CIEDs under close surveillance [32].

## Difficulties faced in MR scanning of CIED and attempts to improve the scanning process

### The need to follow the vendor-specific protocol for each MR conditional device

Each company has their own specific algorithm to program a CIED to ‘MRI-mode’. Common features include disabling bradycardia and tachycardia therapy. Once the patient has been removed from the scanner, the device will be programmed back to pre-scan settings.

Each manufacturer usually provides their own generic check list and algorithm specifics. It is impossible for radiological staff to remember the specific manufacturer-defined safety conditions for every single device.

**Fig. 5:**
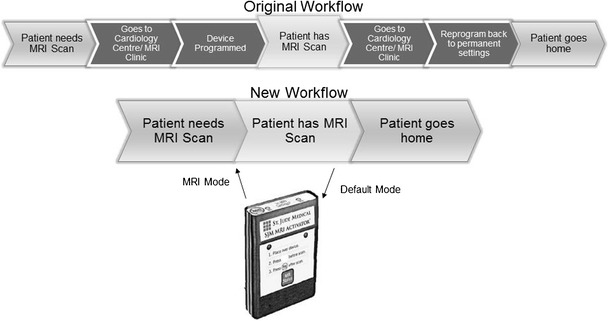
Flowchart demonstrating a streamlined workflow with the incorporation of a hand-held external activator (St Jude Medical, used with permission)

Recently, there have been attempts to simplify and reduce the number of steps within the algorithm and reduce the amount of time the patient remains in MRI mode. The care program pathway by St Jude is a proposed workflow for preparation of patients with pacemakers undergoing MRI. The goal is to minimize the time the patient is required to be in MRI mode. The time the patient needs to be in ‘MRI mode’ can be further reduced by external hand-held activators which simplify access to MRI settings [31, 33] (See Fig. 5).

Key considerations for an effective framework for the safe scanning of CIED patients are:

1. Registry to find out the specific model for the implant
2. Details regarding certain conditions in which the device is MRI-conditional
3. Parameters for scanning which will need to be adjusted for the specific device
4. Re-programming to pre-scan conditions after scanning

Philips has proposed its own method to simplify this process using a mechanism known as “Scanwise”. Scanwise allows automatic scan parameter adjustments to match each specific MR conditional implant. The technology is also able to reduce near metal susceptibility artefacts but this is mainly targeted at orthopaedic implants, and the efficacy in reducing artefacts from CIEDs is unknown [34].

The Sorin group has also its automatic MRI mode exclusively available in its KORA 100 and KORA 250 models, where the device can switch automatically to asynchronous mode when a strong magnetic field is detected. The device switches back 5 min after the patient is removed from the magnetic field [35].

### Coordination and communication with cardiologists and manufacturer cooperation

A cardiologist or pacemaker technician should be present for the device to be switched into MR compatible-mode although this may change in the future. Lack of familiarity or inability to identify the device model may affect decision-making on the type of scan, and a scan may be rejected based on safety grounds. Radiologists should take the initiative to advise referring clinicians when a MRI scan is warranted but has not been considered due to presence of an implant.

Ideally, a central repository by manufacturers for the identification and instructions for use and MRI conditional availability of all their devices, should be created for cardiologists, radiologists and referring clinicians. Another solution is to create a standard industry design platform to allow manufacturers to standardize the process in which MRI-conditional devices are activated. However, availability of different models may result in difficulty in implementing a standardized algorithm, and the issue of propriety systems will further prevent vendors from cooperating to create a one-size-fits-all system.

### Availability and limitations of novel devices

The availability of MRI-compatible cardiac implantable electronic devices varies by country, but this is driven primarily by country-specific regulatory approvals.

In most countries around the world, MRI compatible cardiac devices should be available for use, although specific model availability would differ. For example, the KORA100 and KORA 250 are not available for sale or distribution in the USA.

### Limitations of MR conditional devices

MR-conditional devices must be used within a set of defined parameters for these to function safely during a scan.

Patients must wait 6 weeks after implantation prior to MRI, and many of these devices are not recommended within a field strength above certain field strengths. Pre-existing devices, old leads and pulse generators must be removed for replacement if a new pacemaker is to be inserted.

Certain CIED models cannot be scanned with the iso-center over the thorax, although newer models have no zonal restriction. Some devices still require specialized personnel and monitoring.

Lastly, there is increased cost compared to conventional devices. Therefore, MR-conditional CIEDs are often more favourable for younger patients or in those who are more likely to require MR studies in their lifetime [36].

## Pictorial review of the radiographic features of MR-conditional devices

Plain radiographs may be used to identify the pacemaker as a MR-conditional device as there are manufacturer and model-specific markers. In cases where the device model cannot be read due to positioning, certain features of the markers and components may be used to indicate if a CIED is MR-conditional [37]. Unfortunately, these markers are unique to each manufacturer and conditions prior to scanning differ from model to model.

In the rare occasion where the patient’s implantation records are unavailable, it is often useful to be able to visually identify if a device is MR-compatible. The following is a pictorial review of the different types of CIEDs and methods to identify the model and MR compatibility across several manufacturers.

**Fig. 6:**
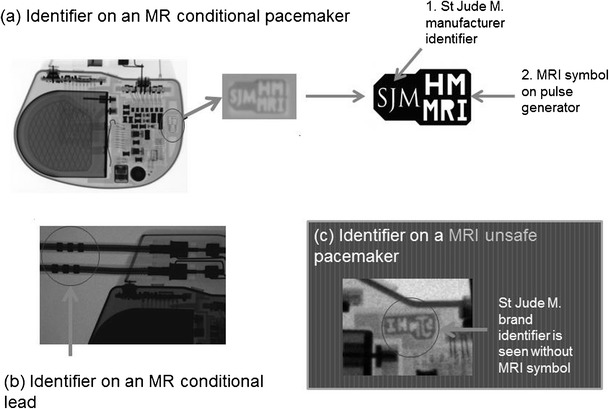
Radiographic images (magnified) demonstrate identifier labels on **a** a MR conditional pacemaker (Accent™ MRI, used with permission) **b** MR conditional lead (Tendril™, used with permission) **c** MRI unsafe pacemaker (Accent™, used with permission)

**Fig. 7:**
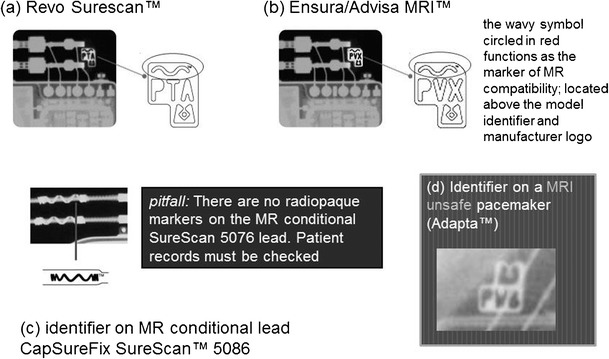
Magnified radiographic images demonstrate Medtronic manufacturer specific markers (images used with permission). **a-b** pacemakers which are MR conditional **c** MR conditional lead **d** MRI unsafe pacemaker identifier

**Fig. 8:**
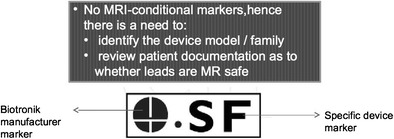
Enhanced radiographic image of the radiopaque marker for the Biotronik EviaTM device (image used with permission). This device has no specific marker to show that it is MR-conditional; hence, there is a need to identify the device model and family

**Fig. 9:**
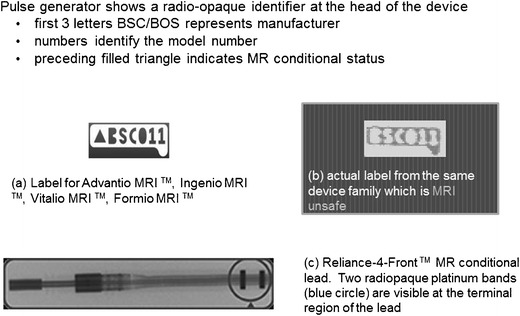
Magnified radiographic images demonstrate radiopaque identifiers from BostonScientific devices **a** MR conditional devices **b** MRI unsafe devices and **c** MR-conditional lead. (images used with permission)

**Fig. 10:**
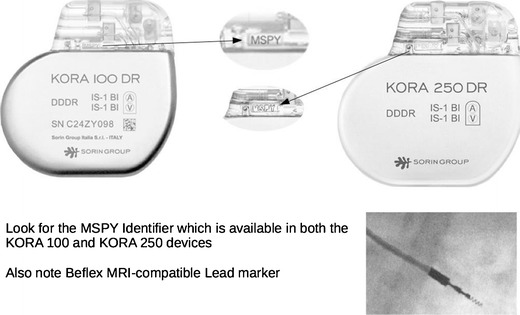
Sorin Group MRI compatible pacemakers. Look for the MSPY identifier. (images used with permission)

**Fig. 11:**
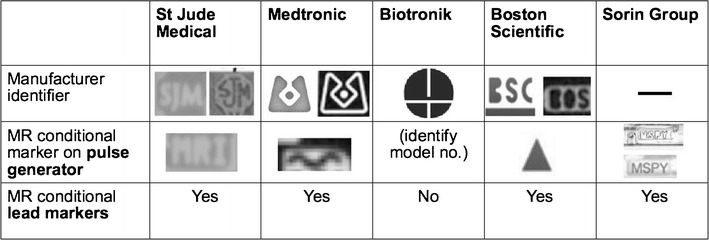
Table demonstrates magnified device radiopaque markers from various manufacturers

- St Jude [37] (See Fig. 6)

- Medtronic [38] (See Fig. 7)

- Biotronik (See Fig. 8)

- Boston Scientific (See Fig. 9)

- Sorin Group [36] (See Fig. 10)

- Summary diagram (See Fig. 11)

- MR conditional implantable cardioverter-defibrillator (ICD) [37, 39]

**Fig. 12:**
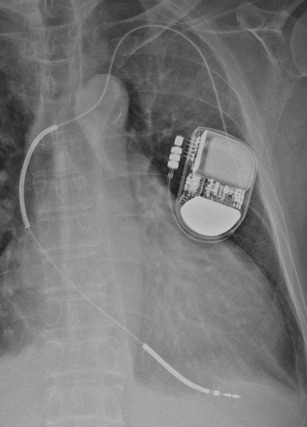
Chest radiograph demonstrating an AICD in situ. A limited number of AICDs are MR-conditional, such as the Medtronic Evera™ MRI ICD and BioTronik ProMRI® AICD

This is radiographically similar to pacemakers apart from high-voltage defibrillation coils which appear as thick bands at the SVC and RV apex (See Fig. 12).

- Subcutaneous ICDs (SICD)

A SICD has no transvenous leads and does not make contact with the heart. The pulse generator is usually located along the left lateral chest wall.

**Fig. 13:**
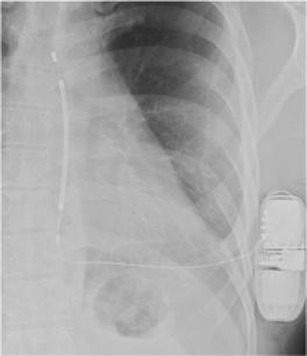
The subcutaneous electrode is implanted in the left parasternal position. There is currently only one manufacturer with this device (Cameroon Health/Boston Scientific)

A study of 22 examinations (15 patients) showed no evidence of tissue injury, device malfunction or interaction with programmed parameters during MR scanning. Unlike transvenous ICDs, heating of the electrode does not harm the myocardium but may cause severe discomfort. Even so, more data is required to support the SICD as a MRI conditional device (See Fig. 13) [40].

- Implantable loop recorders (ILR)

**Fig. 14:**
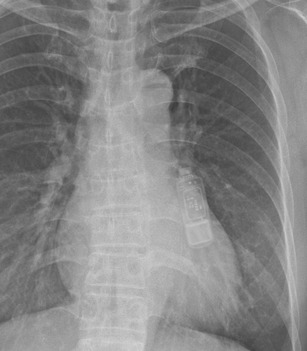
A single-lead right-sided pacemaker is present. There is also an abandoned left-sided lead without the pulse generator unit

This is a leadless device with the appearance of a “USB stick” or cricket-bat. The device is MR-conditional, although device memory/ECG recording will be inaccurate during scanning. Artefacts may mimic arrhythmia, and this should be taken into consideration. Prior to scanning, the patient should be warned about a tugging sensation (See Fig. 14) [41].

- Permanent leadless pacemakers (PLP)

PLPs are implanted within the heart, often within the right ventricle. These are not detectable on physical examination and may not be sensed by a metal detector. Recently, MRI-compatible devices have been released into the market [42].

- Abandoned leads

**Fig. 15:**
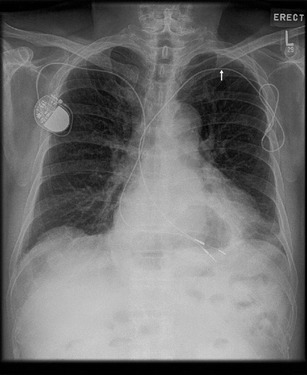
A single-lead right-sided pacemaker is present. There is also an abandoned left-sided lead without the pulse generator unit, indicated by an arrow.

A single-lead right-sided pacemaker is present. There is also an abandoned left-sided lead without the pulse generator unit, indicated by an arrow (See Fig. 15).

An abandoned lead is disconnected from the pulse generator, left behind due to fracture, insulation breaks, dislodgment or other failure. Langman DA, et al. showed that abandoned leads exhibited increased lead tip heating compared to pacemaker-attached leads [43]. Even if the lead is MR compatible, MRI is not recommended.

## Algorithm and protocols

### MRI-conditional algorithm

**Fig. 16:**
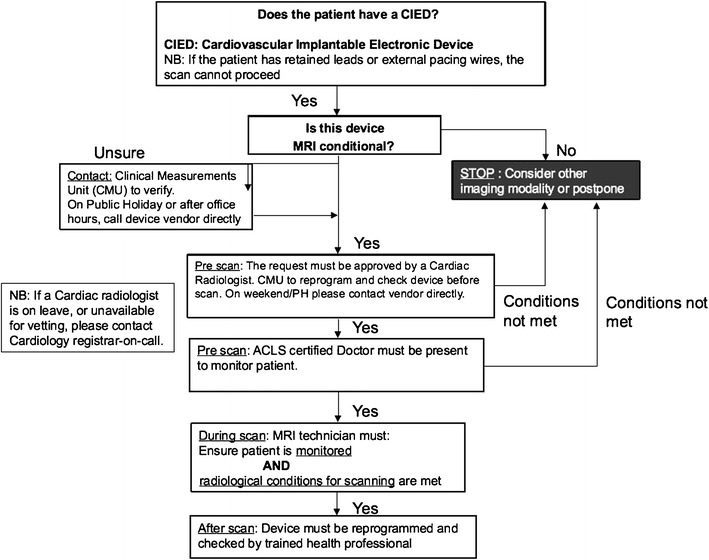
Algorithm for the MR scanning of patients with CIEDs

An algorithm has been developed in our institution to ensure that patients with CIEDs can be MR scanned safely. The first two steps of this algorithm include a mechanism in-built into the hospital computerized order entry system where the requesting clinicians also are required to complete a set of checklists as a part of the patient’s care pathway (See Fig. 16).

### Proposed inadvertent scanning prevention algorithm

**Fig. 17:**
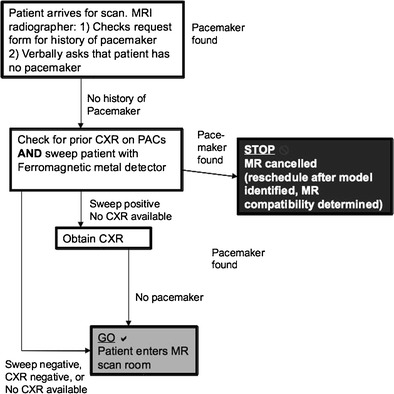
Algorithm for the prevention of inadvertent scanning of patients with CIEDs (Both MR-conditional and non-MR conditional)

Another algorithm was developed in our institution to prevent inadvertent scanning of patients with CIEDs. Three fundamental actions are incorporated (See Fig. 17):

• checking clinical history

• checking for prior chest radiograph

• sweeping with a metal detector.

### Proposed contingency plan after inadvertent scanning

**Fig. 18:**
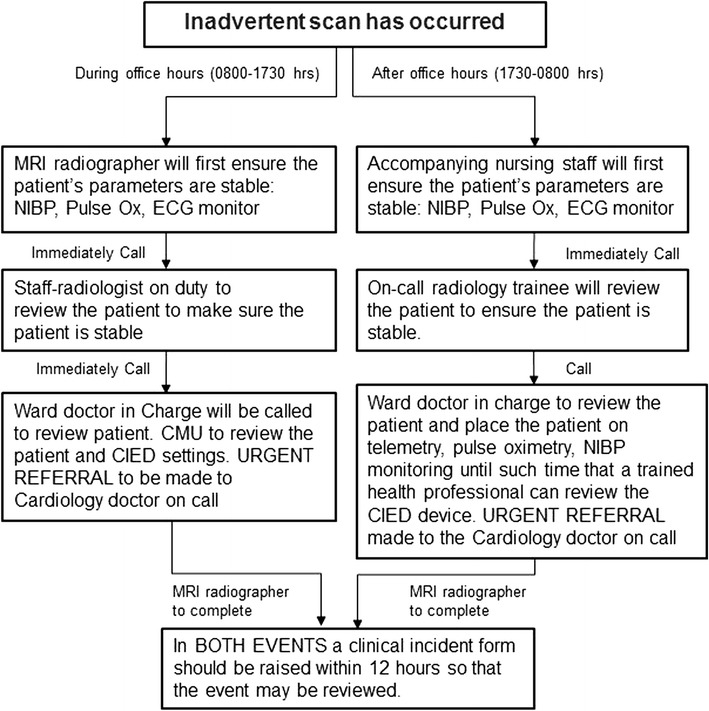
Algorithm after inadvertent scan of patient with CIED

We propose a contingency plan used in our institution should an inadvertent MR scan of a patient with a MRI unsafe CIED occur (See Fig. 18).

## Conclusion

There is an increasing number of patients with CIEDS who will require MRI. Radiologists must be able to facilitate the safe utilization of MRI in patients who have CIEDs.

Note: Previous related electronic exhibit presented at: American Roentgen Ray Society Annual Meeting 2014, Toronto [44].
